# Health impacts of a remotely delivered prolonged nightly fasting intervention in stressed adults with memory decline and obesity: A nationwide randomized controlled pilot trial

**DOI:** 10.1017/cts.2024.651

**Published:** 2024-11-11

**Authors:** Dara L. James, Chung Jung Mun, Linda K. Larkey, Edward Ofori, Nanako A. Hawley, Kate Alperin, David E. Vance, Dorothy D. Sears

**Affiliations:** 1 Edson College of Nursing and Health Innovation, Phoenix, AZ, USA; 2 Department of Psychiatry and Behavioral Sciences, Johns Hopkins School of Medicine, Baltimore, MD, USA; 3 College of Health Solutions, Arizona State University, Phoenix, AZ, USA; 4 Department of Psychology, College of Arts and Sciences, University of South Alabama, College of Liberal Arts and Sciences, Mobile, AL, USA; 5 Barrett Honors College, Arizona State University, Tempe, AZ, USA; 6 School of Nursing, University of Alabama Birmingham, Mobile, AL, USA

**Keywords:** Behavior change, feasible interventions, dietary strategy, health outcomes, remote trials

## Abstract

**Objective/Goals:**

Cognitive decline is intricately linked to various factors such as obesity, stress, poor sleep, and circadian rhythm misalignment, which are interrelated in their impact on cognitive health. Irregular food-intake timing further compounds these issues. The practice of prolonged nightly fasting (PNF) may help synchronize food intake with circadian rhythms, potentially mitigating adverse effects of cognitive decline and associated factors.

**Methods:**

A pilot nationwide, remotely delivered, 2-arm randomized controlled trial was conducted to assess the 8-week outcomes of cognition, stress, and sleep, after a PNF intervention (14-hr nightly fast, 6 nights/week, no calories after 8 pm) compared to a health education (HED) control condition. Participants were living with memory decline, stress, and obesity and had weekly check-in calls to report fasting times (PNF) or content feedback (HED).

**Results:**

Participants were enrolled from 37 states in the US; *N* = 58, 86% women, 71% white, 93% non-Latinx, mean (SD) age 50.1 (5.1) years and BMI 35.6 (3.6) kg/m^2^. No group differences existed at baseline. Linear mixed-effects models were used to compare outcome change differences between groups. Compared to the HED control, the PNF intervention was associated with improved sleep quality (*B* = −2.52; *SE* = 0.90; *95% CI* −4.30–−0.74; *p* = 0.006). Perceived stress and everyday cognition significantly changed over time (*p* < 0.02), without significant difference by group.

**Discussion:**

Changing food intake timing to exclude nighttime eating and promote a fasting period may help individuals living with obesity, memory decline, and stress to improve their sleep. Improved sleep quality may lead to additional health benefits.

## Introduction

There are approximately 6.7 million people in the United States living with Alzheimer’s disease (AD). By 2050, this number is anticipated to reach approximately 13 million [1]. AD and Alzheimer’s disease related dementias (ADRD) have significant short- and long-term impacts on patients, family, and friends. AD/ADRD has profoundly detrimental effects on quality of life, ability to live and function independently, financial status and burden, emotional and physical capabilities, and long-term health trajectory, potentially contributing to decreased life expectancy. The gravity of AD/ADRD on a population scale is particularly pronounced considering that the prevalence of AD/ADRD is expected to double in less than 40 years [2]. This public health challenge intersects significantly with obesity. It is becoming clear that obesity is a contributing risk factor for age-related cognitive decline. Obesity is associated with deficits in multiple cognitive domains, for example, attention, decision-making, memory, and executive function [3], the development of mild cognitive impairment (MCI), and risk of progression to more aggressive neurodegenerative diseases (e.g., AD/ADRD) [4]. More than one-third of the U.S. population is living with obesity; by 2030, greater than 50% of the U.S. population is expected to be living with obesity [5].

Stress is a multifaceted risk factor for both cognitive impairment and obesity. Longitudinal studies demonstrate that perceived stress is associated with impaired cognitive function [6–8]. Stress also significantly reduces sleep quality and duration, which may negatively impact on cognitive function [9]. Stress contributes to the risk of obesity by triggering unhealthy eating behaviors, such as post-dinner nighttime food intake, selection of nutrient-poor, energy-dense foods (e.g., high fat/sugar) [10], and episodic binge eating [11]. Thus, stressed adults living with or at elevated risk for obesity represent an important population to study, as they face challenges that can exacerbate the detrimental effects of obesity on cognitive health.

Intermittent fasting, as a dietary strategy and modifiable lifestyle intervention, has garnered significant attention in the literature and media for its potential benefits on myriad health outcomes. Cross-sectional and small-scale intervention studies of intermittent fasting have demonstrated improvements in weight loss, glucoregulation and lipid profiles, inflammation [12], sleep [13], and metabolic biomarkers associated with type 2 diabetes, cardiovascular disease, and cancer [14]. Less work has been conducted on the impact of intermittent fasting on cognitive function. Preclinical mice studies suggest that intermittent fasting may improve cognitive function by increasing the production of brain-derived neurotrophic factor (BDNF) [15], a protein involved in the growth and survival of neurons, reduction of brain inflammation [16], and promotion of autophagy [17]. Time-restricted feeding (e.g., daily 16-hour fasting and 8-hour feeding) without calorie restriction reduced AD pathology and gene expression related to neural inflammation, and improved sleep and cognition in two mouse models of AD [18]. These preclinical studies suggest that intermittent fasting activates processes that can improve neuronal function and protect against and/or slow the progression of neurodegenerative diseases, including AD/ADRD. As an alternative dietary strategy and modifiable lifestyle intervention, intermittent fasting has garnered significant attention for its potential benefits on myriad health outcomes.

Circadian rhythm misalignment, partly triggered by mistimed eating behaviors and/or habits (e.g., late-night eating), can facilitate decreased poor sleep quality, metabolic function, and inflammation [19–21]—important risk factors impacting neurocognitive health, obesity, and stress [22,23]. Prolonged nightly fasting (PNF), a specific subtype of intermittent fasting regimen, emphasizes fasting during the evening and overnight. As PNF involves fasting during low activity and “sleep” phases of the circadian clock it is a promising intervention for promoting circadian alignment and other health improvements in individuals living with obesity and cognitive decline [24,25]. Habitual evening and overnight fasting practices and PNF interventions have demonstrated benefits on glucoregulation and sleep [14], as well as clinical cancer recurrence and cardiovascular outcomes [13,26–28].

No prior studies, however, have explored the potential cognitive benefits of PNF among midlife adults living with stress and obesity. Furthermore, most studies on intermittent fasting have been geographically restricted (e.g., single site) and have lacked an appropriately matched (e.g., time and attention) control group. To address these gaps, we conducted a nationwide, remotely delivered, pilot randomized-controlled trial (RCT) of PNF. This aimed to examine changes in both perceived and performance-based cognitive function (primary outcomes), as well as sleep quality and stress (secondary outcomes) among midlife adults self-reporting cognitive decline, stress, and obesity.

## Materials and methods

### Study design

A two-arm pilot RCT was conducted nationwide as an 8-week study delivered remotely, with data collection occurring at baseline and post-intervention. The study arms included a PNF intervention group and a health education (HED) control group. Baseline and post-intervention data collection were conducted via Zoom with participants and study staff (one-on-one). Prior to study start, the Institutional Review Board approved the study protocol and procedures (IRB ID: STUDY00015014); the study was registered through ClinicalTrials.gov (NCT05388318). All participants provided written informed consent.

This pilot study was funded through the Institute for Social Science Research at Arizona State University.

### Participants

Enrolled participants (*N* = 58) were recruited through the NIH-sponsored ResearchMatch online platform. From March 2022 through May 2023, 174 participants were screened for eligibility. Participant eligibility was based on the following eligibility criteria. Inclusion criteria included: (1) 40–59 years old; (2) living with obesity (BMI 30 kg/m^2^ to 45.9 kg/m^2^, calculated by study staff with self-report height and weight); (3) moderately-to-severely stressed (Perceived Stress Scale-4 [PSS-4] total score ≥ 5); (4) responded “yes” to the eligibility question “Do you feel your memory is getting worse?;” (5) was willing to be randomized into one of two study groups; (6) was able to speak and read English; (7) had access to a computer and Wi-Fi; (8) had a smart phone; (9) had an at-home weight scale; and (10) lived within the United States. Exclusion criteria included: (1) previously diagnosed with AD or other type of dementia, psychological, psychiatric, or neurological diseases; (2) already routinely fasting for 12+ hours a night; (3) had a prior diagnosis of a clinical eating disorder; (4) had a diagnosis of diabetes and/or any medically based reason that would preclude them from safely participating in fasting; (5) worked a night shift job; (6) prior bariatric surgery; (7) currently pregnant or breastfeeding; and (8) currently engaged in formal weight loss program.; or (9) responded “no” the to the eligibility question: “Do you feel your memory is getting worse?”

### Sample size/power analysis

Given the nature of the current pilot study design, a power analysis to determine sample size was not conducted prior to study start. The sample size for this pilot study was based on our previous work using the same PNF intervention in a single-group pilot study. The current pilot RCT serves as a further step in this line of research to support conducting a large-scale clinical trial.

The design of the HED control group was intended to match the PNF intervention group with respect to time and attention, and to ensure that the HED control group was different enough from the PNF intervention group and did not include fasting related content. Participants across both groups received the same interaction time with the study staff during the intervention (i.e., weekly check in calls to report on study engagement).

### PNF intervention

Participants in the intervention group were asked to engage in the 8-week PNF intervention specified as six days of the PNF protocol with one “day off” (i.e., did not need to fast) per week. The specific “day off” was based on participant preferences and could change weekly as desired (i.e., did not have to be the same day each week). Participants were asked to begin their 14-hour nightly fast no later than 8:00 pm, and to follow the fast with a 10-hour daytime eating window. During the fast, participants could drink water, coffee, or tea (without dairy/alternative dairy products or sweeteners/artificial sweeteners). Participants were free to choose the 10-hour eating window that worked best for them, conditional to their fast start time of no later than 8:00 pm, and their fast lasting for 14 hours overnight. Additionally, participants were asked to “eat as they normally would” (i.e., no changes to diet quantity or quality). Via email, participants were provided with weekly log sheets to write down their start and stop PNF times for each day, including their weekly “day off.” One-on-one check-in calls were conducted once weekly between the study staff and participants (lasting approximately 10 minutes) during which the participant reported their weekly PNF start and stop times and shared their thoughts regarding the intervention including barriers and facilitators.

### HED control group

Participants in the HED control group were asked to engage in an 8-week health education program. Each week, participants assigned to the HED control group were asked to watch 10–15 minutes of YouTube videos that focused on health education topics not related to diet or fasting. Weekly topics were as follows: *Week 1*: Sleep hygiene, *Week 2*: Sun safety, *Week 3*: Home safety, *Week 4*: Driving safety, *Week 5*: Hydration, *Week 6*: Dental health, *Week 7*: Working environment, and *Week 8*: Communication. Participants were asked to watch the videos at their leisure prior to each weekly check-in. One-on-one check-in calls (via phone lasting approximately 10 minutes) were conducted once weekly between the study staff and participants during which they were to confirm that they had watched the videos and provide feedback on the content. The design of the HED control group was intended to match the PNF intervention group for time and attention, and to ensure that the HED control group was different enough from the intervention group and did not include related content (e.g., diet). Participants across both groups received the same interaction time with study staff (i.e., weekly check in calls to report on study engagement).

### Randomization

A covariate-adaptive allocation procedure, carried out using the randPack package in R version 3.4.3, was used to ensure balance in sample size across groups by key predictors of BMI and PSS-4 scores.

### Procedures

The primary recruitment strategy was the use of the online NIH- ResearchMatch platform which aims to connect individuals interested in participating in research with current/ongoing research studies. Email batches were sent to individuals registered through ResearchMatch who met the filtered criteria of: (1) age (40–59 years old) and (2) BMI ≥ 30 kg/m^2^ to 45.9 kg/m^2^. Other eligibility criteria were unavailable options for filtering on ResearchMatch. Once prospective participants indicated interest through their ResearchMatch account, study, staff received their contact information. A follow-up email was sent including additional study information and next steps to schedule an eligibility screening phone call.

Eligibility screening occurred via a brief phone call (approximately 10 minutes) with study staff and was scheduled at participants’ day/time preference. Verbal consent (from the potential participant to the study staff member) was given prior to the eligibility screening process, and informed consent was then signed prior to enrolling in the study. Eligibility screening procedures were structured such that this step of the screening process occurred via phone prior to engagement in the research intervention; there were minimal risks to individuals. This process was approved by the IRB and did not violate individuals rights or welfare. Individuals who were eligible and interested in joining the study were then emailed an informed consent form to sign and return to study staff via email.

Baseline and post-intervention data (i.e., self-reported measures and the objective measurement of cognitive function) were collected remotely via one-on-one Zoom meetings with participants and study staff. All data were entered directly into REDCap (Research Electronic Data Capture) [29,30] by study staff. Additional qualitative data were collected for the PNF group only at post-intervention; a separate manuscript reporting the qualitative exit interview results is currently in preparation.

### Measures

#### Demographics

Participant age, race, ethnicity, gender, sex, marital status, employment, education, insurance status, living situation, and income were collected at baseline. Participants were asked if they had a family history of MCI, AD, or ADRD.

### Primary outcomes

#### Telephone montreal cognitive assessment (T-MoCA)

The T-MoCA is a remote performance-based validated screening tool for cognitive impairment. The T-MoCA separately evaluates five cognitive function domains: (1) memory, (2) attention, (3) language, (4) abstraction, and (5) orientation. A composite score is computed by summing the five cognitive function domain scores. The maximum T-MoCA total score is 22 points, with higher scores indicating better cognitive performance [31].

#### Everyday cognition-12 (E-Cog-12)

The E-Cog-12 was used to determine the extent of functional changes in major daily activities associated with cognitive decline [32]. Participants were asked to rate changes in their perceived cognitive function on a 4-point Likert-type scale. These ratings were subsequently averaged to generate for a global composite score ranging from 1 to 4, with higher scores indicating greater cognitive impairment. Cronbach’s *α* at baseline was 0.85.

### Secondary outcomes

#### The Pittsburgh sleep quality index (PSQI)

[33] was used to measure subjective sleep quality over the previous month. PSQI includes seven subscale components: sleep quality, sleep latency, sleep duration, habitual sleep efficiency, sleep disturbances, use of sleep medications, and daytime disturbances. PSQI contains 19 items yielding a global score ranging from 0 to 21; higher scores indicate poorer global sleep quality. A PSQI global score of > 5 is considered indicative of significant sleep disturbance [34]. Cronbach’s α at baseline was 0.70.

#### Perceived stress scale-10 (PSS-10)

Perceived stress was measured with the Perceived Stress Scale-10 (PSS-10) [35,36]. The PSS-10 measures the degree to which respondents consider their life to be “unpredictable, uncontrollable, and overloading” over the previous month. PSS-10 is scored on a 5-point Likert-type scale ranging from 0 = never to 4 = very often. Total scores range from 10 to 40; higher scores indicate higher stress levels. The PSS-10 showed good internal consistency (Cronbach’s *α* = 0.87).

#### Engagement and adherence

Engagement was measured for both the PNF intervention and HED control conditions by counting a participant’s completed weekly check-ins with study staff (via email, phone, or text) during the 8-week study period. Adherence to the PNF intervention protocol was measured in three ways: (1) Perfect adherence to calorie intake cessation by 8:00 pm was calculated using the reported number of PNF days when fasting started by 8:00 pm divided by the expected total number of PNF days across 8-weeks. The total expected number of PNF days was 48, given one “day off” allowed per week. (2) Perfect adherence to the target fasting duration was calculated using the number of PNF days when the fasting interval reported was at least 14 hours divided by the expected 48 PNF days. (3) Near-perfect adherence to the target fasting duration was calculated using the number of PNF days when the fasting interval reported was at least 13 hours divided by the expected 48 PNF days. With respect to adherence, our focus was on the PNF intervention group and we therefore did not measure adherence to the HED control group; moving forward this is a measure that could be included to better understand the differences/similarities between the control and intervention groups.

### Data analytic plan

Chi-square and t-tests were conducted to compare baseline sociodemographic and clinical characteristics between PNF and HED groups. Engagement and adherence results were presented using descriptive statistics. To address *a priori* hypotheses, several sets of analyses were conducted under an intent-to-treat (ITT) framework. Specifically, a linear mixed-effects model with time (i.e., pre- and post-treatment) nested within participants was employed to test the interaction between time and intervention group for each outcome. This model tests differential treatment effects on rate of change from pre- to post-treatment in outcomes. For all models, random variation in the initial status of the outcome (i.e., random intercept) was allowed. Each model was fitted via restricted maximum likelihood (REML). Linear mixed-effects model allows for inclusion of full range of data that was collected multiple times without discarding data and is robust to missing data [37]. The *lme4* package from R was used to conduct linear mixed-effects models.

## Results

### Sample characteristics

**Figure 1.**
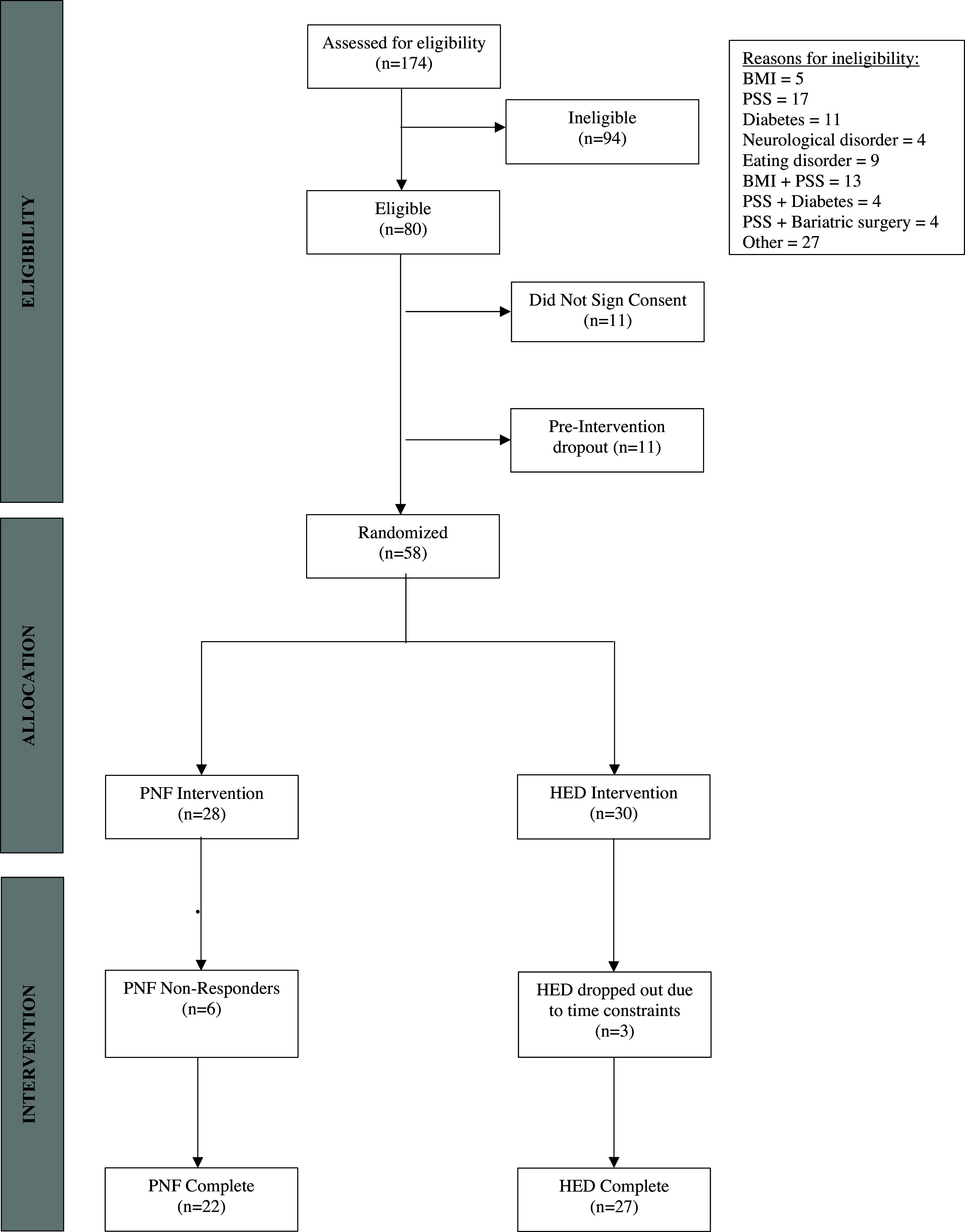
CONSORT participant flow chart. PNF = prolonged nightly fasting; HED = health education control: BMI = body mass index; PSS = perceived stress score.

A total of 174 participants were screened for eligibility. Of these, 94 were ineligible, 80 were eligible and 58 chose to enroll and were randomized into either the PNF intervention group (*N* = 28) or the HED control group (*N* = 30) (see Figure 1 for participant flow chart). Throughout the study duration, in total nine participants dropped out and 49 participants (76.2% sample retention) completed the study (PNF: *N* = 22; HED: *N* = 27); six participants dropped out of the PNF intervention group and three participants dropped out of the HED control group. Participants were enrolled from 37 states across the U.S.

Table 1 shows the participant demographic and clinical characteristics at baseline. No significant group differences were detected. The participants were living with obesity, had an average age of 50 years, with the majority being non-Hispanic White women who were married, employed full-time, had an annual income exceeding $75,000, and possessed high levels of education. About one-third of the participants reported a family history of MCI, AD, or ADRD. The baseline mean T-MoCA total score was suggestive of MCI.

**Table 1.** Baseline demographics and clinical characteristics

		Overall (*N* = 58)	PNF (*N* = 28)	HED (*N* = 30)	*P* value
**Demographics**					
**Age**		50.1 (5.1)	50.1 (5.1)	50.1 (5.2)	>0.9
**Sex**					0.6
	Female	86%	82%	90%	
	Male	14%	18%	10%	
	Declined to respond	0%	0%	0%	
**Race**					0.5
	White	71%	71%	70%	
	American Indian/ Alaskan Native	3.4%	0%	6.7%	
	Black or African American	14%	14%	13%	
	Asian	8.6%	11%	6.7%	
	Native Hawaiian or other Pacific Islander	1.7%	3.6%	0%	
	Unsure/ Declined to respond	1.7%	0%	3.3%	
**Ethnicity**					0.5
	Hispanic/Latinx	6.9%	3.6%	10%	
**Years of Education**		16.3 (2.9)	16.4 (2.4)	16.2 (3.3)	0.9
**Annual Household Income**					>0.9
	Less than $10,000	10%	11%	10%	
	$10,000–$14,999	5.2%	3.6%	6.7%	
	$15,000–$24,999	5.2%	7.1%	3.3%	
	$25,000–$34,999	10%	7.1%	13%	
	$35,000–$49,999	19%	18%	20%	
	$50,000–$74,999	21%	25%	17%	
	$75,000–$99,999	28%	29%	27%	
	>$100,000	1.7%	0%	3.3%	
**Marital Status**					0.5
	Married	57%	54%	60%	
	Divorced	19%	14%	23%	
	Widowed	3.4%	3.6%	3.3%	
	Never been married	21%	29%	13%	
**Employment Status**					>0.9
	Full-time	53%	54%	53%	
	Part-time	16%	14%	17%	
	Unemployed	31%	32%	30%	
**Family history of MCI, AD, or ADRD**		28%	25%	30%	0.9
**Clinical Characteristics**					
**BMI**		35.6 (3.6)	35.4 (3.2)	35.9 (4.0)	0.6
**T-MoCA total score**		18.6 (2.4)	18.5 (2.8)	18.7 (2.1)	0.7
**PSQI total score**		8.7 (3.9)	9.7 (4.1)	7.9 (3.5)	0.1
**PSS total score**		17.9 (6.0)	18.3 (6.7)	17.6 (5.4)	0.7
**ECog-12 mean score**		1.9 (0.6)	2.0 (0.6)	1.8 (0.5)	0.3

PNF = prolonged nightly fasting; HED = health education control; MCI = Mild Cognitive Impairment, AD = Alzheimer’s Disease, and ADRD = Alzheimer’s Disease Related Dementias; BMI = body mass index; T-MoCA = Telephone Montreal Cognitive Assessment; PSQI = Pittsburg Sleep Quality Index; PSS = Perceived Stress Scale; ECog-12 = Measurement of Everyday Cognition.

### Findings of linear mixed-effects ITT analysis

**Figure 2.**
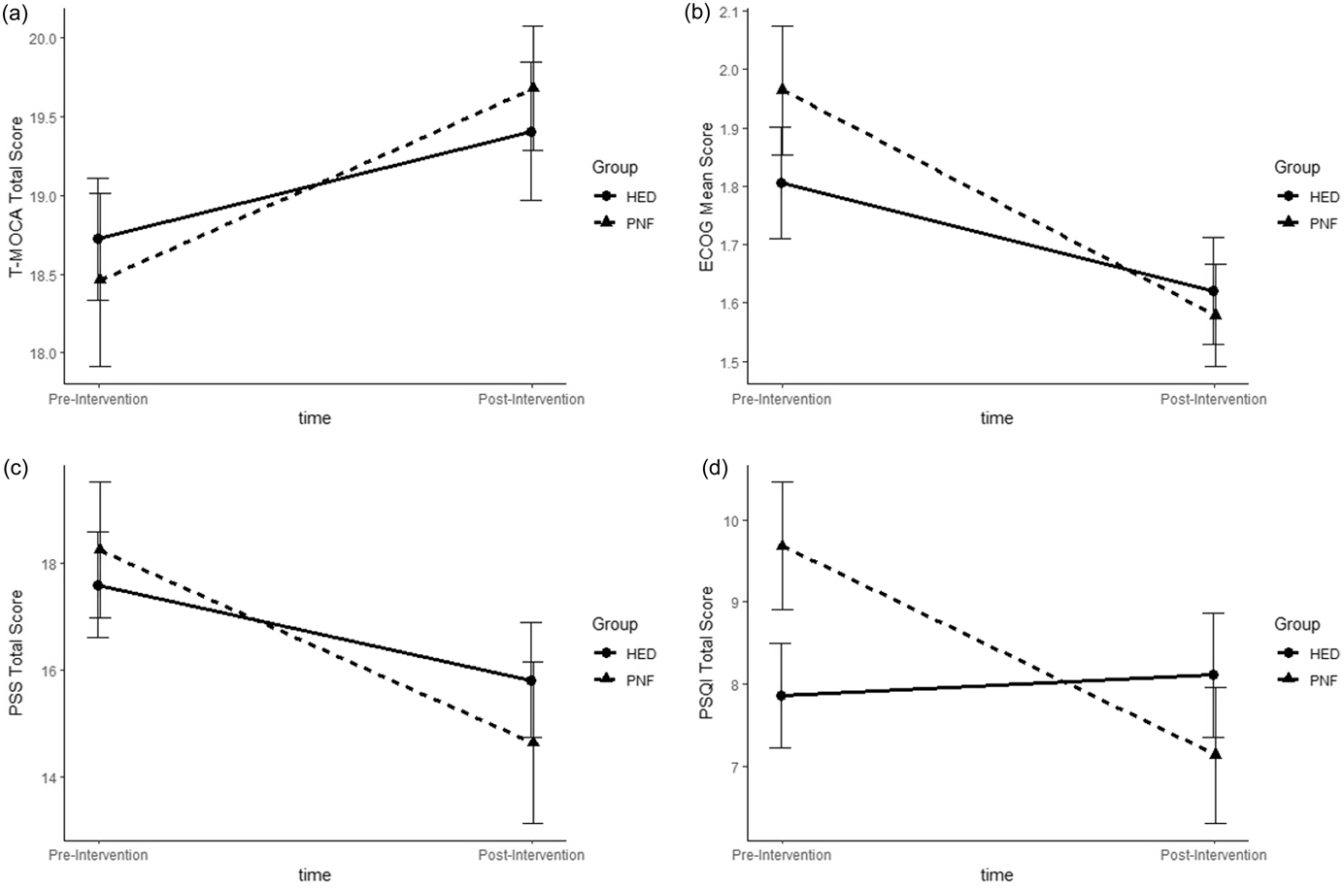
Pre- and post-treatment changes in outcomes by treatment group. The data presented represent means and standard errors. T-MoCA = telephone montreal cognitive assessment, ECog-12 = everyday cognition-12, PSS = perceived stress scale-10, PSQI = Pittsburgh sleep quality index.

Table 2 summarizes the linear mixed-effects models for study outcomes that were tested based upon the ITT framework. There were no significant differences between the PNF and HED groups in terms of changes in primary outcomes: cognitive functioning measured by T-MoCA and self-reported cognitive functioning measured by E-COG-12 (see Figures 2a,b). Regarding secondary outcomes, while there was no significant difference between the PNF and HED groups in changes in perceived stress measured by PSS-10 (see Figures 2c), sleep quality measured by PSQI showed a significant time by group interaction (see Figure 2d). This interaction effect indicates that the PNF intervention group experienced a greater decrease in sleep quality disturbance compared to the HED group from pre- to post-intervention, by an average of 2.52 points on the PSQI scale. With respect to effect sizes (Cohen’s *d*) measured at post-treatment, T-MoCA (*d* = 0.13), E-COG-12 (*d* = 0.05), and PSS-10 (*d* = 0.12) exhibited overall small effects in favor of the treatment, while PSQI (*d* = 0.32) showed a small-to-moderate effect.

**Table 2.** Summary of mixed-effects ITT analysis for primary and secondary outcomes

	Primary Outcomes								Secondary Outcomes							
	T-MoCA				ECog-12				PSS				PSQI			
	*B*	*SE*	*95% CI*	*p*	*B*	*SE*	*95% CI*	*p*	*B*	*SE*	*95% CI*	*p*	*B*	*SE*	*95% CI*	*p*
Intercept	18.72	0.43	17.88 –19.57	**<0.001**	1.81	0.09	1.62 – 1.99	**<0.001**	17.60	1.12	15.38 – 19.82	**<0.001**	7.87	0.70	6.47 –9.26	**<0.001**
Group (0 = HED, 1 = PNF)	−0.26	0.62	−1.49 –0.97	0.672	0.16	0.14	−0.11 –0.43	0.245	0.65	1.61	−2.54 –3.84	0.687	1.81	1.01	−0.20 –3.82	0.077
Time (0 = Pre, 1 = Post)	0.66	0.52	−0.27 –1.60	0.164	−0.21	0.07	−0.35 –-0.07	**0.005**	−1.87	0.76	−3.38 –-0.35	**0.016**	0.34	0.61	−0.87 –1.55	0.580
Time × Group	0.49	0.70	−0.90 –1.87	0.488	−0.15	0.11	−0.36 –0.07	0.174	−0.98	1.14	−3.23 –1.28	0.392	−2.52	0.90	−4.30 – -0.74	**0.006**

HED = Health Education Control group, PNF = Prolonged Nightly Fasting group, Pre = Pre-Intervention, Post = Post-Intervention, T-MoCA = Telephone Montreal Cognitive Assessment, ECog-12 = Everyday Cognition-12, PSS = Perceived Stress Scale-10, PSQI = Pittsburgh Sleep Quality Index.

### Engagement and adherence

Engagement was measured across the total study population and separately by group. Overall, 100% of participants completed six or more of the eight possible weekly check-ins; 96% of participants completed seven or more check-ins and 88% completed all eight. Among the PNF intervention group, 100% of participants completed six or more check-ins; 90% of participants completed seven or more check-ins and 73% completed all eight. Among the HED control condition, 100% of participants completed six or more check-ins; 96% of participants completed seven or more check-ins and 89% completed all eight.

Adherence to the fasting protocol was assessed in the PNF intervention group. Adherence to starting the nightly fast no later than 8:00 pm was 94.8%. On average, participants completed at least 14 hours of fasting for 45 of 48 days (93.8% adherence). Thirteen participants (59.0%) fasted for 14 hours for all 48 days and fourteen (63.6%) participants fasted for 13 hours for all 48 days. In a prior longitudinal analysis of nightly fasting in breast cancer survivors, a 13-hour nightly fast was associated with significant benefits in glycemic control, sleep, and breast cancer outcomes; [26] hence, we also evaluated the close-to-perfect adherence to PNF of at least 13 hours in our study.

### Adverse events

No adverse events or effects related to the intervention were reported from either the PNF or HED groups.

## Discussion

The current pilot RCT examined the impact of PNF on cognitive function, sleep quality, and perceived stress among adults living with obesity, memory decline, and stress. We observed no significant difference between the PNF and HED groups on objective and subjective cognitive function (primary outcomes) or perceived stress (a secondary outcome). However, the PNF group demonstrated a small-to-moderate, significant improvement in sleep quality (a secondary outcome) compared to the HED group. Our overall sample retention rate (76.2%) was considered acceptable, especially considering that the study was conducted remotely across the U.S. among a population of stressed adults. Study engagement and adherence were also high.

The non-significant intervention effects of PNF on cognitive function are somewhat consistent with findings from two recent clinical trials that did not find significant benefits of intermittent fasting in improving cognitive function. In a small pilot study (*N* = 10), Anton and colleagues [38] reported that time-restricted feeding (i.e., fasting for 16 hours per day for 4 weeks) was not effective in improving cognitive function, as measured by standard MoCA, among older adults with overweight. In an RCT among women who were overweight (*N* = 51), Teong and colleagues [39] showed that intermittent fasting (i.e., 24 hours of fasting on 3 non-consecutive days per week for 8 weeks) led to significant improvement in the Digit Symbol Substitution Test, measuring processing speed and attention, but not in the Psychomotor Vigilant Task, assessing vigilance and alertness. It should be noted that all the studies, including ours, had small sample sizes and included participants lacking clinically defined cognitive impairment inclusion criteria, and were short-duration interventions, potentially lacking sufficient statistical power to detect small effects of intermittent fasting interventions on cognitive function. All these studies, including ours, were conducted with a non-clinical population, likely resulting in ceiling effects as participants may have had little room for improvement. Nonetheless, it is worth noting that findings from an AD mouse model [18] show remarkably positive effects of time-restricted feeding (i.e., fasting during inactive/sleep phase of the day) on cognitive function and related mechanistic outcomes. This highlights the importance of further clinical research into human nightly fasting and its effects on cognitive function.

The significant improvement in sleep quality, with a small-to-moderate effect size, associated with the PNF intervention is noteworthy. A wealth of literature suggests that sleep issues (e.g., insomnia, sleep disordered breathing, etc.) are important risk factors that often precede and contribute to the development of neurodegenerative disorders (e.g., AD and ADRD) [40,41]. In fact, both animal and human studies have reported that the broad category of sleep issues can adversely impact key neurocognitive processes, including glymphatic clearance, neuroinflammation, and oxidative stress—processes intricately linked to the pathophysiology of neurodegenerative diseases [40,42,43]. Hence, the observed enhancement in sleep quality suggests the potential therapeutic value of PNF in mitigating associated risks. Recent time-restricted feeding studies in AD mouse models also demonstrate significant sleep benefits [18]. However, a recent review of emerging clinical trials suggested that the effects of intermittent fasting on sleep are mixed, and notably, none of the studies incorporated more objective measurements of sleep, such as actigraphy and the polysomnography (PSG) sleep assessment [44]. PSG is not feasible in free-living, larger sample size studies. Actigraphy-derived sleep quality data, collected from research-grade wearable devices, is comparable to PSG. Future studies should incorporate both subjective and objective sleep assessments, and further align on specific sleep domains and outcomes, to more comprehensively evaluate the effects of PNF on sleep outcomes.

### Limitations

There are several limitations to this pilot RCT. First, the sample size was based on our previous pilot studies and did not include sample size calculation or a power analysis; the small sample size (*N* = 58) limits the statistical power to detect small effects and generalizability of findings. Further, it is possible that the small sample size may have impacted the nonsignificant findings are due to the small sample size and not that the intervention doesn’t have an effect on cognition. Second, the study only included individuals who were able to speak and read English. Third, the inclusion criterion for cognitive decline relied on self-report data. Fourth, the intervention length was restricted to 8 weeks, and no follow-up assessments were conducted post-treatment. Extending the intervention period beyond 8 weeks and including follow-up assessments could provide additional insights into the long-term outcomes of PNF. Fifth, we did not collect data on disability status and/or physical activity, both of which could have been confounding variables. While we did collect data about income, we recognize that the largest majority of the population (28%) had an high range of $75,000–$100,000 annually. As the study was conducted fully remotely, the measurement of subjective memory decline (at inclusion) and of adherence and engagement (post-intervention) were solely based on participant self-reporting, and may therefore limit the generalizability of the study findings. Future studies should incorporate objective measures of fasting adherence, such as using continuous glucose monitors. Additionally, the T-MoCA [31], designed as a rapid screening measure for mild cognitive impairment, may not have been sensitive enough to capture subtle changes over a relatively short period. Future studies should include more comprehensive performance-based cognitive testing and explore biological markers (e.g., neurofilament light chain protein) associated with cognitive impairment. Lastly, sleep assessment relied on self-report data. Future studies should incorporate objective measures of sleep and circadian rhythm, such as actigraphy and PSG assessments.

## Conclusion

To our knowledge, this nationwide pilot RCT is the first to examine the effects of PNF on cognitive function, sleep quality, and perceived stress, compared to a HED control condition among a sample of adults living with obesity and self-reported memory decline and stress. No significant differences between the PNF and HED control groups emerged on cognitive function change (primary outcome). Significant difference in improved sleep quality (a secondary outcome) was observed in the PNF group compared to the HED group. No significant group difference was observed in perceived stress change (a secondary outcome). We observed acceptable sample retention and robust adherence to the PNF protocol throughout the 8-week intervention. PNF participants reported no adverse effects. Future studies with larger sample sizes, objective sleep and fasting adherence tracking measures, and comprehensive cognitive testing are warranted to further explore the efficacy of PNF on our outcomes of interest herein. PNF is a non-pharmacological, low burden and cost, lifestyle intervention that may be suitable for a variety of populations to improve health outcomes.
