# Health impacts of a remotely delivered prolonged nightly fasting intervention in stressed adults with memory decline and obesity: A nationwide randomized controlled pilot trial – ADDENDUM

**DOI:** 10.1017/cts.2024.693

**Published:** 2025-01-07

**Authors:** Dara L. James, Chung Jung Mun, Linda K. Larkey, Edward Ofori, Nanako A. Hawley, Kate Alperin, David E. Vance, Dorothy D. Sears

A note regarding the co-first authorship of this article was omitted from the original publication.

Chung Jung Mun should have been noted as the co-first author alongside Dara L. James.
